# Network Pharmacology-Based Investigation of the System-Level Molecular Mechanisms of the Hematopoietic Activity of Samul-Tang, a Traditional Korean Herbal Formula

**DOI:** 10.1155/2020/9048089

**Published:** 2020-02-13

**Authors:** Ho-Sung Lee, In-Hee Lee, Sang-In Park, Dae-Yeon Lee

**Affiliations:** ^1^The Fore, 87 Ogeum-ro, Songpa-gu, Seoul 05542, Republic of Korea; ^2^Forest Hospital, 129 Ogeum-ro, Songpa-gu, Seoul 05549, Republic of Korea; ^3^Forestheal Hospital, 173 Ogeum-ro, Songpa-gu, Seoul 05641, Republic of Korea

## Abstract

Hematopoiesis is a dynamic process of the continuous production of diverse blood cell types to meet the body's physiological demands and involves complex regulation of multiple cellular mechanisms in hematopoietic stem cells, including proliferation, self-renewal, differentiation, and apoptosis. Disruption of the hematopoietic system is known to cause various hematological disorders such as myelosuppression. There is growing evidence on the beneficial effects of herbal medicines on hematopoiesis; however, their mechanism of action remains unclear. In this study, we conducted a network pharmacological-based investigation of the system-level mechanisms underlying the hematopoietic activity of Samul-tang, which is an herbal formula consisting of four herbal medicines, including *Angelicae Gigantis Radix*, *Rehmanniae Radix Preparata*, *Paeoniae Radix Alba*, and *Cnidii Rhizoma*. *In silico* analysis of the absorption-distribution-metabolism-excretion model identified 16 active phytochemical compounds contained in Samul-tang that may target 158 genes/proteins associated with myelosuppression to exert pharmacological effects. Functional enrichment analysis suggested that the targets of Samul-tang were significantly enriched in multiple pathways closely related to the hematopoiesis and myelosuppression development, including the PI3K-Akt, MAPK, IL-17, TNF, FoxO, HIF-1, NF-kappa B, and p53 signaling pathways. Our study provides novel evidence regarding the system-level mechanisms underlying the hematopoiesis-promoting effect of herbal medicines for hematological disorder treatment.

## 1. Introduction

Hematopoiesis refers to the process of development of immature precursor cells into various mature and functional blood cells, which initiates from the self-renewing multipotent hematopoietic stem cells (HSCs) [1, 2]. This biological process involves accurate coordination of cellular proliferation, differentiation, and survival of progenitor cells to maintain hematopoietic homeostasis modulated by the activity of various cytokines, growth factors, and key regulatory factors, as well as the complex interactions between hematopoietic cells, tissues, and organs [1, 2]. However, hematopoietic homeostasis disruption caused by various reasons, including anticancer therapies (e.g., chemotherapy and radiotherapy), myeloid malignancies, nutritional deficiencies, or viral infection, may lead to the development of hematological disorders (HDs) such as myelosuppression [3–5]. Myelosuppression is a pathophysiological condition characterized by reduced bone marrow ability to produce sufficient amounts of blood cells (e.g., erythrocytes, leukocytes, and thrombocytes), which results in immunodeficiency, anemia, leukocytopenia, neutropenia, and thrombocytopenia. [3–5]. Symptoms of myelosuppression include fatigue, headache, fever, infection, bruising, shortness of breath, excessive bleeding, pain, and diarrhea, which might affect the quality of life and could be life-threatening if not effectively managed [4–9]. The current pharmacological strategies for treating myelosuppression involve the administration of erythropoietin (EPO), granulocyte colony-stimulating factor (G-CSF), and granulocyte-macrophage colony-stimulating factor (GM-CSF) [10–12]; however, they have been reported to cause unfavorable side effects such as bone and muscle pains, fever, flushing, and nausea [13, 14]. This indicates the need to develop therapies for effective HD amelioration with improved safety. Herbal medicines, which are characterized by multicomponents, multitarget, and multipathway pharmacological mechanisms [15–17], have attracted considerable attention and are recognized as effective therapeutic agents for the myelosuppression alleviation; further, they have fewer side effects than conventional therapies [18–24]. There is growing evidence on the beneficial effects of various herbal medicines in terms of promotion and enhancement of hematopoiesis *in vitro* and *in vivo* [25–33].

Samul-tang (Si-wu-tang; SMT) is an herbal formula comprising four herbal medicines, including *Angelicae Gigantis Radix* (*Angelica gigas*; AGR), *Rehmanniae Radix Preparata* (*Rehmannia glutinosa*; RRP), *Paeoniae Radix Alba* (*Paeonia lactiflora*; PRA), and *Cnidii Rhizoma* (*Cnidium officinale*; CR) [34–36]. It is commonly used to treat various HDs and related symptoms such as anemia [37], dysmenorrhea [38–40], chemotherapy-induced myelosuppression [41, 42], and menstrual disorders [43]. Previous studies have reported that the hematopoietic effects of SMT partly involve the modulation of cellular processes in bone marrow cells, HSCs, and blood cells (e.g., erythrocytes, leukocytes, and thrombocytes), as well as the activities of key hematopoietic factors (e.g., EPO, G-CSF, interleukins (ILs), and interferon- (IFN-) *γ*) [42, 44–46]. However, the system-level molecular therapeutic mechanisms of SMT are yet to be fully elucidated.

Network pharmacology is an interdisciplinary science that aims to uncover the pathophysiological mechanisms underlying various diseases and their treatment strategies at a system-level by integrating biomedicine, pharmacology, systems biology, network biology, computational science, and other related scientific fields [16, 47, 48]. This interdisciplinary approach has been shown to be useful for discovering active compounds contained in herbal drugs and their corresponding potential targets, and investigating the therapeutic mechanisms that involve complex interactions between multiple compounds and targets, which may facilitate the exploration of the pharmacological properties of herbal medicines [47, 48]. In this study, we conducted a network pharmacology-based investigation of the system-level molecular mechanism underlying the hematopoietic activity of SMT.

## 2. Materials and Methods

### 2.1. Investigation of Chemical Compounds Contained in SMT

We retrieved the chemical compounds present in the four herbal medicines that constitute SMT (i.e., AGR, RRP, PRA, and CR) from traditional Chinese medicine (TCM)-related databases, including the Traditional Chinese Medicine Systems Pharmacology (TCMSP) database, Traditional Chinese Medicine Integrated Database (TCMID), and Herb Ingredients' Targets (HIT) database [49–52]. Regarding AGR, we first explored the biochemical constituents of *Angelicae Sinensis Radix*, a commonly used herbal medicine in China that belongs to the *Angelicae* species, using the aforementioned TCM-related databases, and combined those with the reported major compounds of AGR such as marmesin, lomatin, and decursin [53–55]. Chemical compounds not contained in AGR, including isoeugenol, stigmasterol, and 4-octanone, were excluded from the integrated data based on previous studies [53–55].

### 2.2. Exploration of Active Compounds of SMT

To investigate the potential bioactive chemical compounds in SMT, we explored the absorption, distribution, metabolism, and excretion (ADME) properties of each individual phytochemical compound present in the four herbal constituents of SMT. In this study, we assessed the three commonly used ADME-related parameters (oral bioavailability (OB), Caco-2 permeability, and drug-likeness (DL)) for each compound [52]. OB indicates the fraction of an ingested dose of a given drug that crosses the gastrointestinal epithelium, enters the systemic circulation, and becomes available for distribution to internal tissues and organs [52, 56]. Caco-2 permeability is used to assess the absorption capacity of drug molecules and chemical compounds in the intestines based on their passage rate through the Caco-2 human colon epithelial cancer cell line [52, 57–59]. Notably, Caco-2 cells are commonly used as a model for evaluating the intestinal absorption capacity of biochemical compounds since they have morphologic features similar to those of human intestinal epithelial cells [57–59]. Generally, compounds with Caco-2 permeability <−0.4 are regarded as impermeable in the small intestinal epithelium [60, 61]. DL is a key qualitative criterion used in drug design to determine candidate chemical components that may be used as drugs based on their structural and pharmacokinetic characteristics [52, 62]. Based on previous studies, we regarded chemical compounds with OB ≥ 30%, Caco-2 permeability ≥−0.4, and DL ≥ 0.18 as pharmacologically active [52, 63, 64].

### 2.3. Investigation of the Targets of Active Compounds in SMT

We investigated human target genes/proteins that interact with active phytochemical compounds in SMT using the Search Tool for Interactions of Chemicals (STITCH) 5 [65], Similarity Ensemble Approach (SEA) [66], SwissTargetPrediction [67, 68], and PharmMapper [69]. Computational modeling methods, including systematic drug targeting tool (SysDt) [70] and weighted ensemble similarity (WES) algorithm [71], were also employed for target identification as descried previously [72–78]. Subsequently, we confirmed detailed information regarding the targets, including their scientific name, relevant gene/protein ID, and organism, and further standardized the targets using Uniprot [79]. The myelosuppression-associated human genes/proteins were surveyed using diverse databases, including Online Mendelian Inheritance in Man (OMIM) [80], Therapeutic Target Database (TTD) [81], GeneCards [82], DrugBank [83], Pharmacogenomics Knowledge for Personalized Medicine (PharmGKB) [84], DisGeNET [85], Human Genome Epidemiology (HuGE) Navigator [86], and The Comparative Toxicogenomics Database (CTD) [87] by using search terms for various myelosuppression-related disorders, including “anemia,” “leukopenia,” “neutropenia,” “thrombocytopenia,” “lymphopenia,” “granulocytopenia,” and “agranulocytosis, with the search species limited to “*Homo sapiens*”.

### 2.4. Construction of SMT-Associated Networks

The herb-compound (H-C) and compound-target (C-T) networks were constructed by linking the herbal medicines with their active compounds and the active compounds with their corresponding targets, respectively. The target-pathway (T-P) network was built by linking the targets with their associated signaling pathways. All networks were visualized using Cytoscape software (version 3.7.1) [88]. The target location network was generated based on the analysis of gene expression data of various hematopoietic tissues and organs obtained from The Human Protein Atlas [89] and BioGPS databases [90]; this network was generated by linking the target to the corresponding tissues and organs where it was analyzed to be specifically expressed using previously described procedures [91–98]. The protein-protein interaction (PPI) network was constructed using the STRING database (version 11.0) [99] and interactions with highest confidence scores (≥0.9) were selected for further analysis. In the network, nodes represent the herbal medicines, active phytochemical compounds, targets, or signaling pathways while edges represent the interactions between the nodes [100]. The degree of a node refers to the number of connections it has to other nodes in the network [100].

### 2.5. Functional Enrichment Analysis

Functional enrichment analysis of SMT-targeted genes or proteins was conducted using g:Profiler [101], which is an efficient web server-based tool for the functional profiling of a given list of genes or proteins, and Kyoto Encyclopedia of Genes and Genomes (KEGG) database [102].

## 3. Results

**Figure 1:**
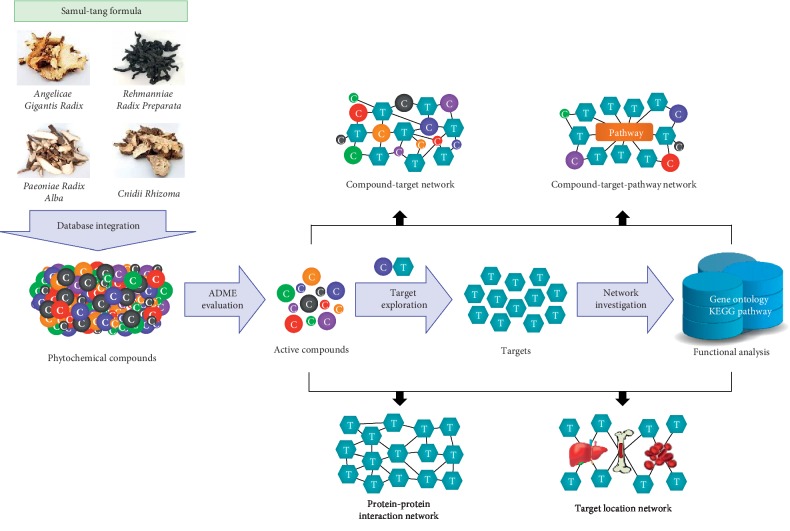
A schematic diagram representing the workflow of the network pharmacology-based investigation of the pharmacological mechanisms of SMT.

The pharmacological mechanisms of SMT were investigated based on the network pharmacology perspective as follows (Figure 1). First, we extensively surveyed the chemical compounds contained in the four herbal medicines that comprise SMT using various TCM-related databases (Figure 1). Next, we assessed ADME parameters, such as OB, Caco-2 cell permeability, and DL, for each individual chemical compound to identify potential bioactive compounds (Figure 1). Subsequently, we determined potential targets of the active chemical compounds by exploring the protein-chemical interactions using relevant databases and conducted functional enrichment analysis of the targets (Figure 1). Furthermore, we merged comprehensive information regarding SMT into the H-C, C-T, T-P, and target location networks and investigated its pharmacological properties based on network pharmacology analysis (Figure 1).

### 3.1. Chemical Compounds of SMT

We investigated the chemical compounds contained in the four herbal medicines (i.e., AGR, RRP, PRA, and CR) that comprise SMT from a number of TCM-related databases (e.g., TCMSP, TCMID, and HIT). Consequently, we obtained 126, 76, 85, and 189 compounds for AGR, RRP, PRA, and CR, respectively, and identified 440 compounds after duplicate removal (Supplementary [Supplementary-material supplementary-material-1]).

### 3.2. Investigation of the Active Phytochemical Compounds of SMT

*In silico* ADME models have been widely used to investigate active phytochemical compounds that may possess therapeutic properties [52, 62]. To determine the potential active compounds of SMT, we evaluated the ADME parameters (i.e., OB, Caco-2 permeability, and DL) of each individual compound contained in the four herbal medicines comprising the herbal formula. As previously described, we considered compounds with OB ≥30%, Caco-2 permeability ≥−0.4, and DL ≥0.18 as pharmacologically active [63, 64]. Moreover, we considered some compounds that did not meet the aforementioned criteria as active components due to their high amount and pharmacological activity. Collectively, 18 active compounds were retrieved for SMT (Supplementary [Supplementary-material supplementary-material-1]).

### 3.3. Identification of the Targets of Active Phytochemical Compounds in SMT

To identify the potential therapeutic targets of SMT, we employed an *in silico* approach to assess the biological interactions between the active phytochemical compounds and human genes/proteins using STITCH 5 [65], SEA [66], SwissTargetPrediction [67, 68], and PharmMapper [69]. Computational modeling methods such as SysDt [70] and WES algorithm [71] were also employed for target identification as descried previously [72–78]. Hence, a total of 230 targets were obtained for the 16 active phytochemical compounds in SMT (Supplementary [Supplementary-material supplementary-material-1]). Note that no potential pharmacological targets were identified for the case of two active compounds (i.e., 11alpha,12alpha-epoxy-3beta-23-dihydroxy-30-norolean-20-en-28,12beta-olide and Paeoniflorin_qt).

### 3.4. Network-Based Analysis of the Pharmacological Mechanisms of SMT

**Figure 2:**
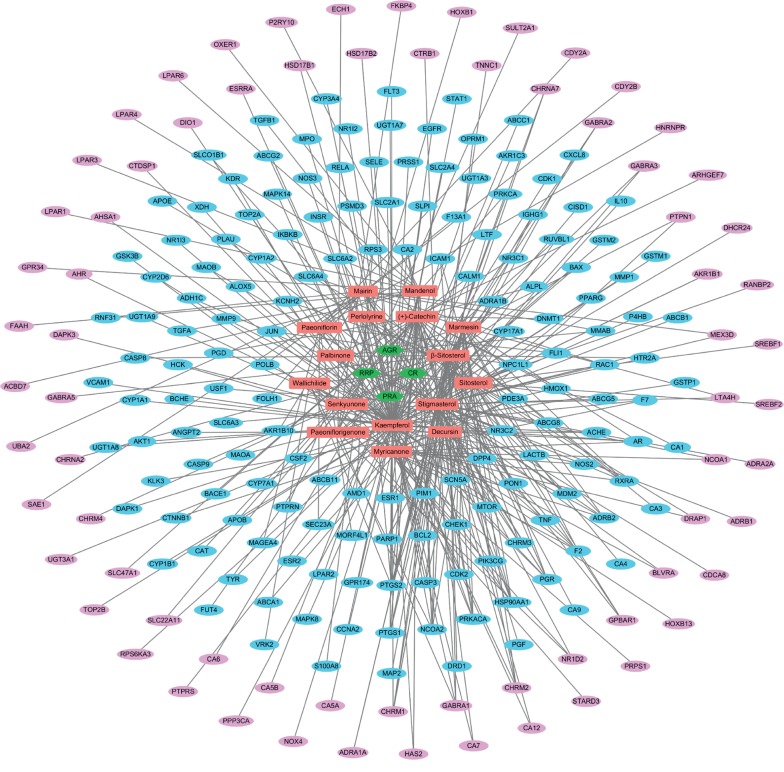
The herb-compound-target network of SMT. Green hexagons and red rectangles indicate the four herbal medicines comprising SMT and their 16 active chemical compounds, respectively. Ovals indicate the 230 targets of the active compounds in SMT, while those closely related to myelosuppression are colored in blue.

**Figure 3:**
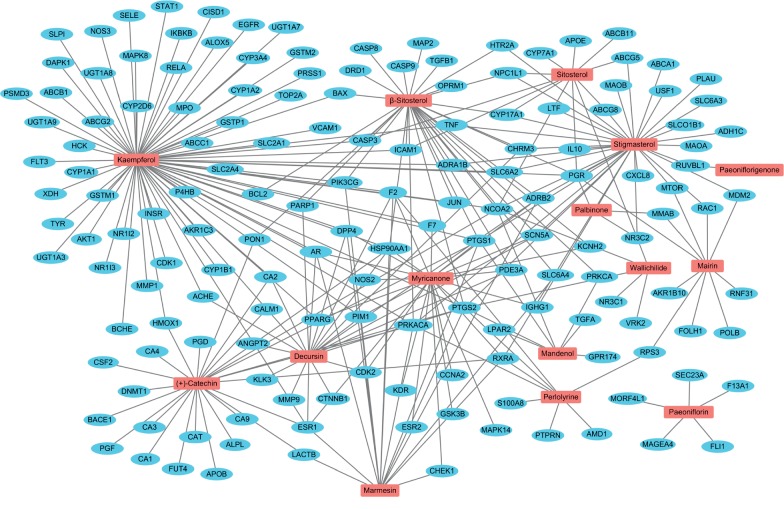
The compound-target network of SMT. Red rectangles and blue ovals indicate the 16 active chemical compounds in SMT and their 158 myelosuppression-associated targets, respectively.

To understand the “multicomponents, multitarget, and multipathway” pharmacological mechanisms of SMT, we constructed an herb-compound-target (H-C-T) network by linking the herbal medicines with the active phytochemical compounds and the active compounds with their potential targets (Figure 2). The H-C-T network for SMT was composed of 250 nodes and 393 edges, including the 4 herbal medicines, 16 active compounds, and 230 targets (Figure 2). Furthermore, for network-based investigation of the system-level therapeutic properties of SMT, we built a C-T network comprised of 173 nodes and 274 edges by linking the active compounds with their myelosuppression-related targets (Figure 3 and Supplementary [Supplementary-material supplementary-material-1]). Of note, none of the targets associated with myelosuppression showed potential interactions with the active compound Senkyunone. The active phytochemical compounds kaempferol (degree = 73), stigmasterol (degree = 34), *β*-sitosterol (degree = 31), and (+)-catechin (degree = 25) had the largest number of connections with the myelosuppression-related targets (Figure 3), which indicated them as the potential primary active compounds responsible for the hematopoietic activity of SMT. Moreover, 53 targets had two or more interactions with the active phytochemical compounds (Figure 3), which demonstrates the multicompound, multitarget pharmacological characteristic of herbal medicines, including SMT.

**Figure 4:**
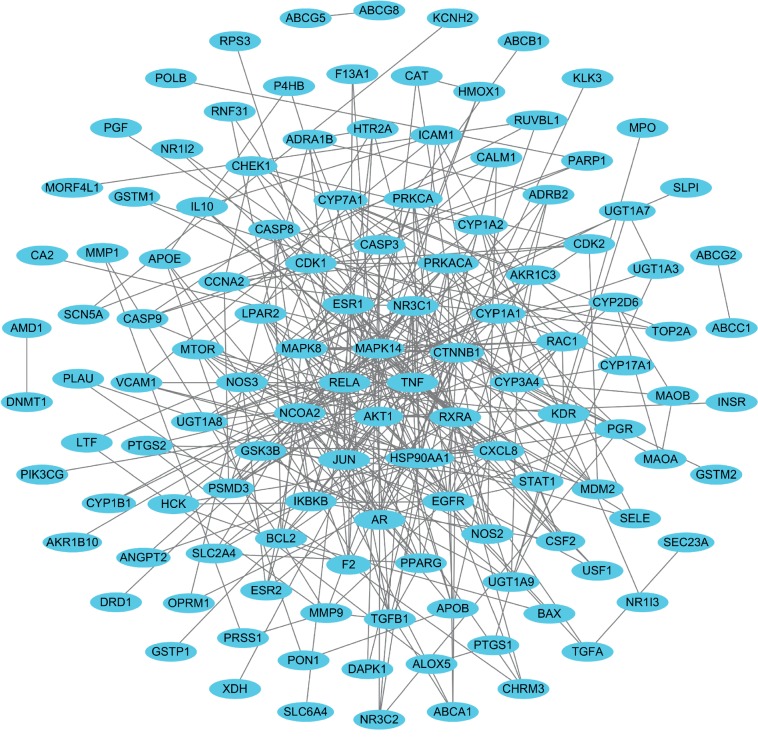
The protein-protein interaction network for myelosuppression-associated targets of SMT. Blue ovals indicate the myelosuppression-associated targets of the active chemical compounds in SMT.

To investigate the underlying relationship between the targets interacting with the active ingredients in SMT, we built a PPI network comprised of 123 nodes and 333 edges for the myelosuppression-associated SMT targets (Figure 4). The centralization and heterogeneity of the PPI network were 0.411 and 2.249, respectively, which suggested that the network may contain hubs, nodes with a large number of interactions [63, 100, 103–105]. Here, a node was determined to be a hub if its degree is greater than or equal to twice the average node degree of the network [106, 107]. Notably, hub nodes have been reported to function as important regulators in a variety of biological processes [108, 109]. Among the myelosuppression-related targets of SMT, AKT1 (degree = 21), TNF (degree = 21), MAPK14 (degree = 19), RELA (degree = 17), JUN (degree = 16), HSP90AA1 (degree = 16), RXRA (degree = 16), CTNNB1 (degree = 15), NR3C1 (degree = 15), ESR1 (degree = 15), MAPK8 (degree = 14), NCOA2 (degree = 13), AR (degree = 13), EGFR (degree = 13), CXCL8 (degree = 12), CYP3A4 (degree = 11), CYP1A1 (degree = 11), and PRKACA (degree = 11) were found to be hub nodes, suggesting that they may play key roles in the hematopoietic activities of SMT (Figure 4). Previous studies have reported a close association between these targets and hematopoiesis or myelosuppression development. For instance, AKT1, TNF, RELA, *β*-catenin (encoded by *CTNNB1*), ESR1, and AR have been reported to serve as important regulators that coordinate HSC function [110–115]. The p38 (encoded by the *MAPK14*) cascade controls the quiescence and expansion of HSCs, which are crucial processes for maintaining hematopoietic homeostasis [116–122]. The p38, JNK1 (encoded by *MAPK8*), and c-JUN (encoded by *JUN*) are essential for erythropoiesis regulation, which is the biological process whereby hematopoietic tissues in the bone marrow produce erythrocytes by modulating proliferation, apoptosis, and differentiation of erythroid cells [123–126]. EGFR promotes HSC regeneration and function after radiotherapy-induced myelosuppressive injury [127]. The cytokine CXCL8 (also known as IL-8) has been reported to stimulate HSC mobilization to exert a radioprotective effect [128].

**Figure 5:**
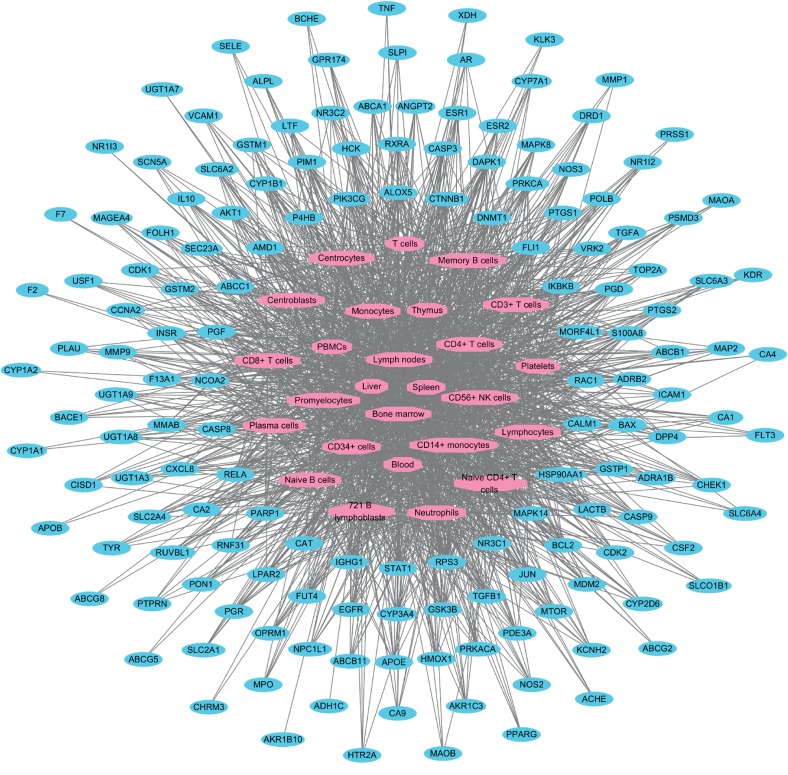
The target location network for myelosuppression-associated targets SMT. Pink octagons represent tissues and organs, while blue ovals represent the myelosuppression-associated targets of the active chemical compounds in SMT.

To understand the therapeutic effects of SMT at the tissue- and organ-level, we generated a target location network based on the gene expression data for individual myelosuppression-associated SMT targets across various tissues and organs, which were identified from The Human Protein Atlas [88] and BioGPS databases [90] and analyzed as previously described [91–98] (Figure 5; see Materials and Methods). Consequently, we found that the targets were expressed in various hematopoietic tissues and organs [129, 130], including the liver (degree = 144), bone marrow (degree = 137), spleen (degree = 116), lymph nodes (degree = 113), and blood (degree = 100) (Figure 5), which suggests the systematic mechanism of action responsible for the pharmacological effects of SMT. Furthermore, all the myelosuppression-associated SMT targets (except ADH1C, AKR1B10, CTNNB1, and CYP17A1) were expressed in two or more tissues and organs, implying that they are closely related to hematopoietic regulation (Figure 5).

Taken together, our findings demonstrate the pharmacological mechanism of action of SMT at a complex network-level.

### 3.5. Functional Enrichment Analysis of the SMT-Associated Network

To explore the functional roles of the myelosuppression-associated targets of SMT, we conducted gene ontology (GO) enrichment analysis of the targets. We found significant enrichment of the targets in GO terms associated with the regulation of diverse biological processes such as hemopoiesis, cell proliferation, cell differentiation, cell cycle process, cell migration, cell apoptosis, immune response, response to iron binding, and inflammation (Supplementary [Supplementary-material supplementary-material-1]), which supports the pharmacological mechanisms of SMT hematopoietic activity.

**Figure 6:**
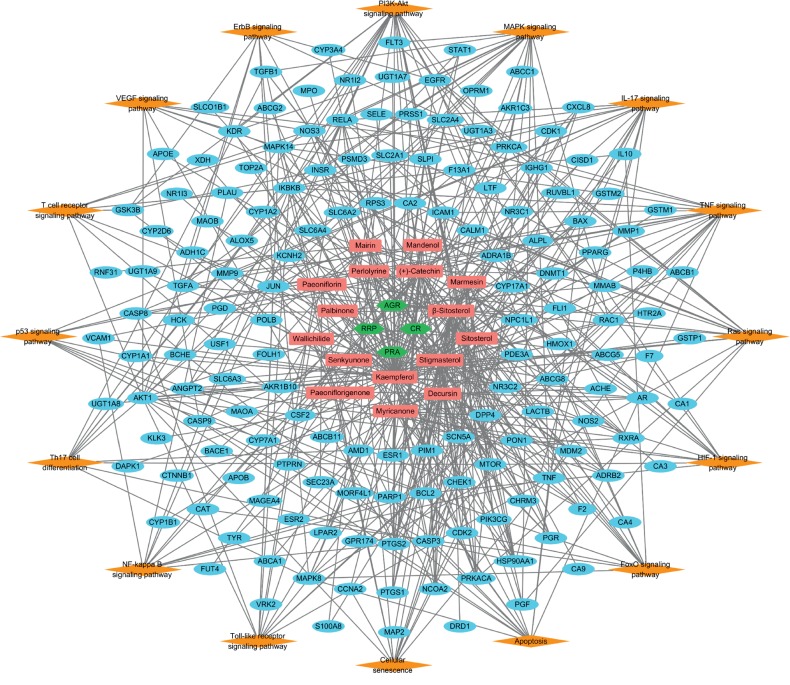
The herb-compound-target-pathway network of SMT. Green hexagons and red rectangles represent the four herbal medicines comprising SMT and their 16 active chemical compounds, respectively. Blue ovals indicate the myelosuppression-associated targets of the active compounds while orange diamonds indicate the signaling pathways enriched with the corresponding targets.

The hematopoietic system is known to be tightly controlled through the precise coordination of various key signaling pathways; further, aberrant regulation of the hematopoiesis-associated pathways could cause various HDs [131–138]. To investigate the pathway-level mechanisms of SMT, we performed KEGG pathway enrichment analysis of its myelosuppression-related targets (Figure 6 and Supplementary Figures [Supplementary-material supplementary-material-1] and [Supplementary-material supplementary-material-1]). As a result, we found that the targets have relatively large number of connections with the “PI3K-Akt signaling pathway” (degree = 23), “MAPK signaling pathway” (degree = 19), “IL-17 signaling pathway” (degree = 16), “TNF signaling pathway” (degree = 15), “Ras signaling pathway” (degree = 15), “HIF-1 signaling pathway” (degree = 12), “FoxO signaling pathway” (degree = 12), “Apoptosis” (degree = 12), “Cellular senescence” (degree = 12), “Toll-like receptor signaling pathway” (degree = 11), “NF-kappa B signaling pathway” (degree = 10), “Th17 cell differentiation” (degree = 10), “p53 signaling pathway” (degree = 9), “T cell receptor signaling pathway” (degree = 9), “VEGF signaling pathway” (degree = 8), and “ErbB signaling pathway” (degree = 8) (Figure 6 and Supplementary Figures [Supplementary-material supplementary-material-1] and [Supplementary-material supplementary-material-1]). A substantial body of research has shown that these highly connected signaling pathways may play important roles in hematopoietic regulation and HD pathogenesis. The PI3K-Akt, MAPK, Ras, FoxO, HIF-1, Toll-like receptor, NF-kappa B, and VEGF pathways are crucial for the functional modulation of the hematopoietic system; further, their aberrant regulation may contribute to HD development [111, 123, 139–163]. The TNF signaling pathway has been reported as a key regulator for the hematopoietic processes by coordinating the HSC function [110, 131, 164, 165]. The p53 signaling pathway-dependent complex interplay between cell cycle control, senescence, and apoptosis is closely involved in the modulation of HSC function and hematopoietic homeostasis [166–171]. Activation of IL-17 (a pro-inflammatory cytokine produced by distinct cluster of differentiation 4^+^ [CD4^+^] T helper 17 [Th17] cells)-associated pathway stimulates granulopoiesis by inducing the proliferation of bone marrow CD34^+^ cells and their differentiation into granulocytes; moreover, IL-17 inhibition might impair hematopoietic recovery and deteriorate myelotoxicity caused by radiation injury [172–182].

We further investigated the functional relationship of the myelosuppression-associated targets of SMT using GeneMANIA [183], a web server for investigating and analyzing functional interactions between multiple genes and proteins based on comprehensive biological data integration. The GeneMANIA analysis indicated that among the targets, 40.62% and 29.88% were predicted to be co-expressed and to have physical interactions, respectively (Supplementary [Supplementary-material supplementary-material-1]), which suggested a similarity of biological functions and activities exerted by the targets.

Collectively, our findings indicate that SMT might exert its therapeutic activities by modulating multiple myelosuppression-associated signaling pathways and relevant cellular processes.

## 4. Discussion

Hematopoiesis is a dynamic developmental process that involves the complex regulation of multiple cellular mechanisms in HSCs, including proliferation, self-renewal, differentiation, and apoptosis, to generate a sufficient number of blood cells required to maintain homeostasis of human physiological functions [1, 2]. Impairment and dysregulation of the hematopoietic system might contribute to the development of various HDs, including myelosuppression [3–5]. There has been increasing attention toward herbal medicines as therapeutic agents for HDs given their effective hematopoietic activities and less side effects [18–23]. In this study, we explored the system-level pharmacological mechanisms underlying the hematopoietic effects of SMT by employing a network pharmacology approach [47, 184]. The following are our key findings: (i) 16 potentially active phytochemical compounds present in SMT may interact with 158 myelosuppression-related targets to exhibit therapeutic activities; (ii) GO enrichment analysis demonstrated that the targets of the active compounds in SMT were involved in diverse hematopoiesis-associated biological processes such as cell proliferation, cell differentiation, cell cycle process, cell migration, cell apoptosis, immune response, response to iron binding, inflammation, and hemopoiesis; (iii) the myelosuppression-associated targets of SMT were significantly enriched in various pathways, including the PI3K-Akt, MAPK, IL-17, TNF, FoxO, HIF-1, NF-kappa B, and p53 signaling pathways, which are associated with the hematopoiesis and HD development.

SMT is comprised of four herbal medicines (i.e., AGR, RRP, PRA, and CR) containing 16 active phytochemical compounds that interact with 158 myelosuppression-related targets as investigated by the network pharmacological approach. These herbal and chemical constituents of SMT have been reported to improve hematopoietic function, and therefore alleviate myelosuppression. AGR and PRA have been reported to ameliorate immunosuppression and hematopoietic dysfunction induced by cyclophosphamide treatment, which is a bone marrow-suppressive cytotoxic alkylating agent [185, 186]. RRP, *β*-sitosterol, and kaempferol are known to possess hematopoietic and immunomodulatory properties *in vitro* and *in vivo* [187–191]. (+)-catechin stimulates the proliferation of bone marrow cells and thereby exerts protective effects against myelosuppression induced by chemotherapeutic agents in mice [192]. Moreover, paeoniflorin has a hematopoietic activity and promotes the recovery of bone marrow function in radiotherapy-induced myelosuppressed mice via the upregulation of G-CSF and GM-CSF [193]. Taken together, these previous findings support the hematopoietic and immunomodulatory effects of the herbal and chemical constituents of SMT.

Previous experimental studies have demonstrated the hematopoietic role of SMT. For instance, SMT has been reported to stimulate spleen colony formation and to suppress radiation- and chemotherapeutic agent-induced hematopoietic cell injury, thereby exerting a hematopoiesis-promoting and myeloprotective effect [42, 194–197]. SMT treatment has also been reported to increase the number of peripheral blood cells and to enhance hematopoietic gene expression, including EPO, G-CSF, CD34, and NF-kappa B, in the bone marrow of the blood-deficiency mice model [44, 196, 198–201]. Furthermore, SMT has been shown to increase the amount of erythrocytes and leukocytes as well as the concentration of hemoglobin and hematocrit in blood, and promote the proliferation, cell cycle progression, and differentiation of bone marrow cells [202, 203]. T cell-mediated immunity was decreased in mice injected with anti-tumor drugs, which was improved by the SMT administration [204]. Further experimental studies are warranted to confirm the therapeutic properties of SMT indicated in this study, which might promote the development of effective herbal medicine-based therapies for treatment of myelosuppression.

## 5. Conclusions

In this study, we explored the system-level pharmacological properties of SMT. Network pharmacology analysis investigated 16 potential active phytochemical compounds of SMT that may interact with 158 myelosuppression-associated targets to exert therapeutic effects. The targets were involved in a variety of hematopoiesis-associated biological processes such as cell proliferation, cell differentiation, cell cycle process, cell migration, cell apoptosis, immune response, inflammation, response to iron binding, and hemopoiesis. We further found that the targets of SMT were enriched in various signaling pathways related to the hematopoiesis and myelosuppression development, including the PI3K-Akt, MAPK, IL-17, TNF, FoxO, HIF-1, NF-kappa B, and p53 signaling pathways. In conclusion, our study provides a novel insight into the synergistic and poly-pharmacological action mechanisms of herbal medicines for the HD treatment.

## Data Availability

The data used to support the findings of this study are included within the article.

## Abbreviations

ADME: Absorption, distribution, metabolism, and excretion
AGR: Angelicae *gigantis radix*
AR: Androgen receptor
CR: *Cnidii rhizoma*
C-T: Compound-target
CTD: The Comparative Toxicogenomics Database
CTNNB1: Catenin beta-1
CXCL8: Chemokine (C-X-C motif) ligand 8
CYP1A1: Cytochrome P450 family 1 subfamily A member 1
CYP3A4: Cytochrome P450 family 3 subfamily A member 4
CD34: Cluster of differentiation 34
CD4: Cluster of differentiation 4
DL: Drug-likeness
EGFR: Epidermal growth factor receptor
EPO: Erythropoietin
ESRA: Estrogen receptor alpha
FoxO: Forkhead box protein O
G-CSF: Granulocyte-colony-stimulating factor
GO: Gene ontology
GM-CSF: Granulocyte-macrophage colony-stimulating factor
H-C: Herb-compound
H-C-T: Herb-target-pathway
HD: Hematological disorder
HIF-1: Hypoxia-inducible factor 1
HIT: Herb Ingredients' Targets
HSC: Hematopoietic stem cell
HSP90AA1: Heat shock protein 90 alpha family class A member 1
HuGE Navigator: Human Genome Epidemiology Navigator
IL: Interleukin
IFN: Interferon
KEGG: Kyoto Encyclopedia of Genes and Genomes
MAPK: Mitogen-activated protein kinase
NCOA2: Nuclear receptor coactivator 2
NF-kappa B: Nuclear factor kappa-light-chain-enhancer of activated B cells
NR3C1: Nuclear receptor subfamily 3 group C member 1
OB: Oral bioavailability
OMIM: Online Mendelian Inheritance in Man
PharmGKB: Pharmacogenomics Knowledge for Personalized Medicine
PI3K: Phosphoinositide 3-kinase
PPI: Protein-protein interaction
PRA: *Paeoniae radix alba*
PRKACA: Protein kinase cAMP-activated catalytic subunit alpha
RELA: v-rel avian reticuloendotheliosis viral oncogene homolog A
RRP: *Rehmanniae radix preparata*
RXRA: Retinoid X receptor alpha
SEA: Similarity Ensemble Approach
SMT: Samul-tang
STITCH: Search Tool for Interactions of Chemicals
SysDT: Systematic drug targeting tool
TCM: Traditional Chinese medicine
TCMID: Traditional Chinese Medicine Integrated Database
TCMSP: Traditional Chinese Medicine Systems Pharmacology
Th17 cells: T helper 17 cells
TTD: Therapeutic Target Database
TNF: Tumor necrosis factor
T-P: Target-pathway
WES algorithm: Weighted ensemble similarity algorithm.

## Supplementary Materials

Supplementary Materials Supplementary Figure S1: functional enrichment analysis of the myelosuppression-related targets of SMT. Supplementary Figure S2: pathway enrichment analysis of the myelosuppression-related targets of SMT. Supplementary Figure S3: functional interaction analysis of the myelosuppression-related targets of SMT. Supplementary Table S1: molecular properties of chemical compounds in SMT. Supplementary Table S2: molecular properties of active chemical compounds in SMT. Supplementary Table S3: list of the targets of active chemical compounds in SMT.
