# Supplementary material for: Network Pharmacology-Based Investigation of the System-Level Molecular Mechanisms of the Hematopoietic Activity of Samul-Tang, a Traditional Korean Herbal Formula
Source: Evid Based Complement Alternat Med. 2020 Feb 13;2020:9048089. doi: 10.1155/2020/9048089 (PMC7040423; doi:10.1155/2020/9048089)
Supplement: Supplementary Materials — Supplementary Figure S1: functional enrichment analysis of the myelosuppression-related targets of SMT. Supplementary Figure S2: pathway enrichment analysis of the myelosuppression-related targets of SMT. Supplementary Figure S3: functional interaction analysis of the myelosuppression-related targets of SMT. Supplementary Table S1: molecular properties of chemical compounds in SMT. Supplementary Table S2: molecular properties of active chemical compounds in SMT. Supplementary Table S3: list of the targets of active chemical compounds in SMT. [file 9048089.f1.pdf]

Supplementary Materials

Network pharmacology-based investigation of the system-level molecular mechanisms of the hematopoietic activity of Samul-tang, a traditional Korean herbal formula

Ho-Sung Lee[1], In-Hee Lee[1], Sang-In Park[2], and Dae-Yeon Lee[1,3,*]

[1]The Fore, 87 Ogeum-ro, Songpa-gu, Seoul 05542, Republic of Korea.

[2]Forestheal Hospital, 173 Ogeum-ro, Songpa-gu, Seoul 05641, Republic of Korea.

[3]Forest Hospital, 129 Ogeum-ro, Songpa-gu, Seoul 05549, Republic of Korea.

[*]Correspondence should be addressed to Dae-Yeon Lee; foresthrnd@gmail.com

Supplementary Figures

	Category	Term	Count	Percent [%]	p-value
Gene ontology	Biological process	Cell activation involved in immune response	19	2.69	3.41E-02
		Cytokine production	20	2.57	3.97E-02
		Hematopoietic or lymphoid organ development	29	3.08	7.94E-06
		Hemopoiesis	27	2.99	5.22E-05
		Immune response-regulating signaling pathway	20	2.97	4.68E-03
		Immune system development	29	2.93	2.41E-05
		Leukocyte activation	34	2.63	1.54E-05
		Leukocyte activation involved in immune response	19	2.71	3.15E-02
		Leukocyte cell-cell adhesion	13	3.83	2.20E-02
		Leukocyte degranulation	16	3.01	4.39E-02
		Leukocyte differentiation	19	3.64	4.06E-04
		Leukocyte homeostasis	7	7.87	2.95E-02
		Lymphocyte homeostasis	6	9.52	4.13E-02
		Myeloid cell differentiation	18	4.31	7.13E-05
		Myeloid leukocyte activation	21	3.23	6.59E-04
		Myeloid leukocyte differentiation	14	6.76	9.66E-06
		Regulation of apoptotic process	53	3.32	8.96E-15
		Regulation of apoptotic signaling pathway	22	5.37	2.80E-08
		Regulation of blood circulation	15	4.98	1.61E-04
		Regulation of cell cycle	30	2.47	5.39E-04
		Regulation of cell death	53	3.03	4.83E-13
		Regulation of cell differentiation	40	2.11	2.77E-04
		Regulation of cell migration	28	2.99	2.80E-05
		Regulation of cell motility	28	2.81	1.07E-04
		Regulation of cell population proliferation	57	3.32	3.26E-16
		Regulation of immune response	31	2.82	1.60E-05
		Regulation of immune system process	43	2.61	1.06E-07
		Regulation of inflammatory response	17	4.57	7.44E-05
		Regulation of ion transport	27	3.88	1.93E-07
		Regulation of leukocyte migration	10	5.24	1.67E-02
		Regulation of metal ion transport	18	4.60	2.56E-05
		Regulation of myeloid cell differentiation	12	4.72	5.93E-03
		Regulation of myeloid leukocyte differentiation	9	7.50	2.54E-03
		Regulation of protein kinase activity	22	2.77	4.30E-03
		Regulation of signal transduction	72	2.14	2.69E-11
		Regulation of signaling	81	2.16	8.97E-14
		Response to chemical	131	2.74	4.15E-46
		Response to cytokine	47	3.89	2.65E-15
		Response to drug	65	6.18	4.03E-35
		Response to growth factor	25	3.37	1.83E-05
		Response to iron ion	5	14.71	2.42E-02
		Response to metal ion	27	7.18	7.67E-14
		Response to toxic substance	39	7.26	1.54E-21
		T cell activation	15	3.23	3.58E-02
		T cell homeostasis	6	15.38	2.36E-03

	Category	Term	Count	Percent [%]	p-value
Gene ontology	Cellular components	Blood microparticle	9	6.29	1.08E-03
		Cytoplasmic part	126	1.30	4.64E-11
		Cytoplasmic vesicle part	32	2.15	2.15E-04
		Cytosol	68	1.34	3.10E-03
		Endoplasmic reticulum membrane	27	2.54	7.75E-05
		Endoplasmic reticulum part	34	2.52	1.80E-06
		Extracellular organelle	43	1.98	1.48E-05
		Extracellular region part	64	1.71	5.90E-07
		Extracellular vesicle	43	1.99	1.44E-05
		Intracellular organelle part	109	1.17	1.37E-04
		Intracellular part	141	0.97	1.92E-02
		Intracellular vesicle	46	1.99	3.59E-06
		Membrane part	88	1.24	1.10E-03
		Organelle part	111	1.16	1.96E-04
		Plasma membrane part	54	1.88	9.64E-07
	Molecular function	Drug binding	48	2.74	1.74E-10
		Enzyme binding	66	3.00	3.46E-18
		Heme binding	14	10.14	1.14E-08
		Ion binding	108	1.73	2.66E-15
		Iron ion binding	10	6.45	7.05E-04
		Kinase activity	22	2.66	2.04E-03
		Kinase binding	26	3.58	5.80E-07
		Metal ion binding	66	1.57	9.84E-05
		Nuclear receptor activity	9	19.15	1.58E-07
		Protein binding	138	1.10	8.70E-05
		Protein kinase activity	21	3.42	6.08E-05
		Protein kinase binding	25	3.86	2.60E-07
		Signaling receptor binding	43	2.60	2.33E-08
		Transcription factor binding	22	3.36	3.90E-05
		Zinc ion binding	24	2.91	1.38E-04
Pathway	KEGG	PI3K-Akt signaling pathway	23	6.55	1.10E-05
		MAPK signaling pathway	19	6.46	1.92E-04
		IL-17 signaling pathway	16	17.58	7.14E-10
		TNF signaling pathway	15	13.64	1.39E-07
		Ras signaling pathway	15	6.52	2.23E-03
		HIF-1 signaling pathway	12	12.00	2.79E-05
		FoxO signaling pathway	12	9.30	4.38E-04
		Apoptosis	12	8.89	7.03E-04
		Cellular senescence	12	7.59	3.47E-03
		Toll-like receptor signaling pathway	11	10.78	2.61E-04
		NF-kappa B signaling pathway	10	10.20	1.24E-03
		Th17 cell differentiation	10	9.62	2.10E-03
		p53 signaling pathway	9	12.50	6.43E-04
		T cell receptor signaling pathway	9	9.00	9.22E-03
		VEGF signaling pathway	8	13.56	1.15E-03
		ErbB signaling pathway	8	9.52	1.53E-02

Supplementary Figure S1. Functional enrichment analysis of the myelosuppression-related targets of SMT. A table showing the result of functional enrichment analysis of the myelosuppression-related targets of SMT.

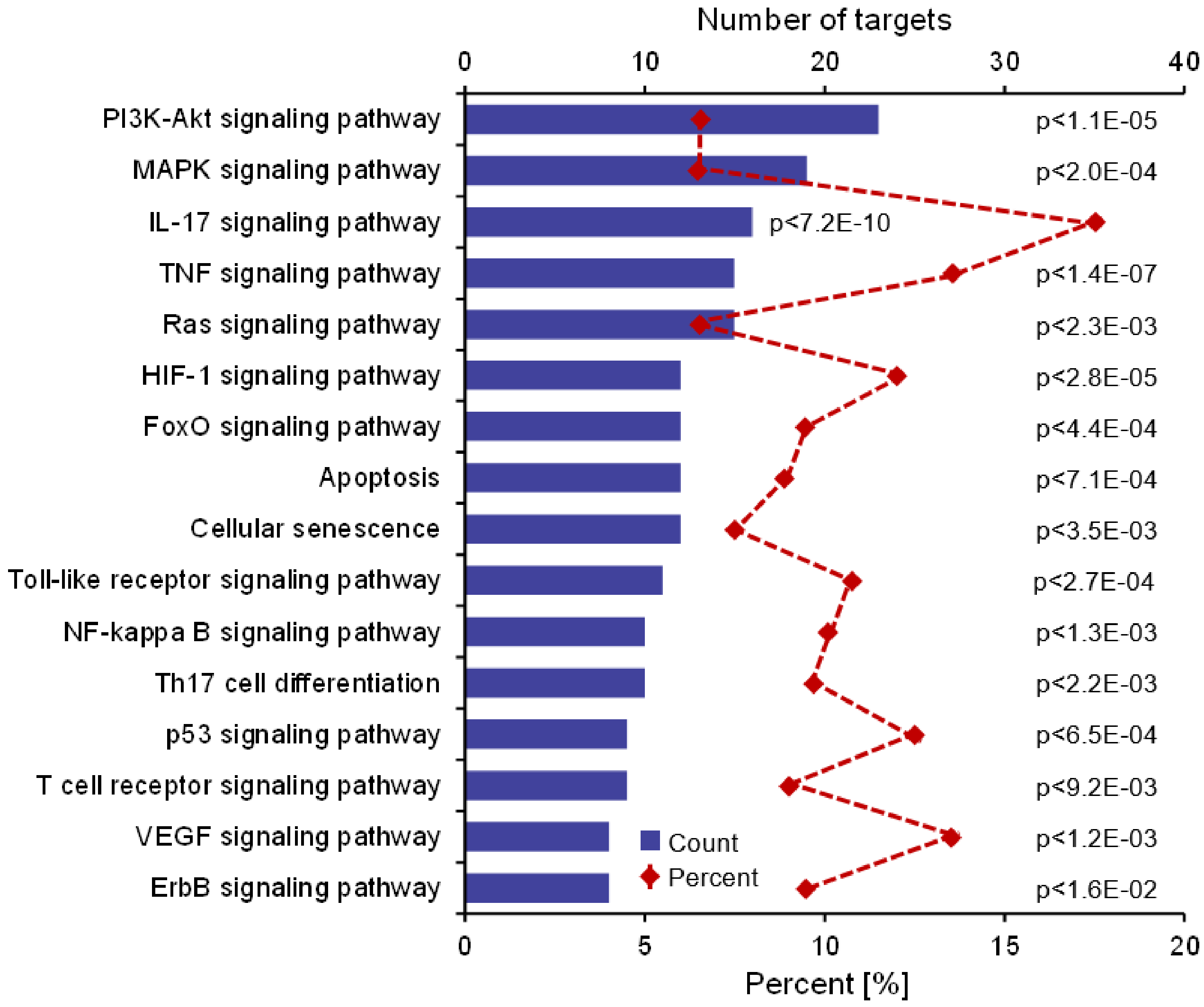

Supplementary Figure S2. Pathway enrichment analysis of the myelosuppression-related targets of SMT. A graph depicting the result of KEGG pathway enrichment analysis of the myelosuppression-related targets of SMT.

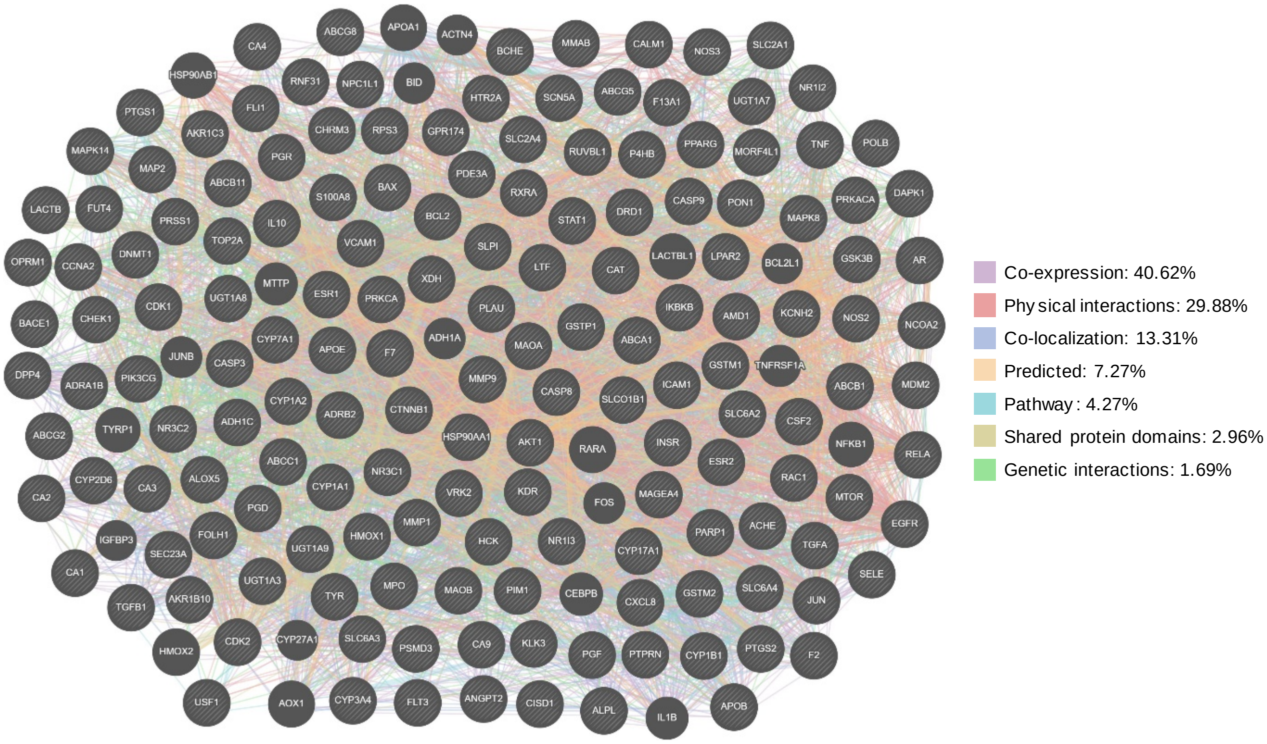

Supplementary Figure S3. Functional interaction analysis of the myelosuppression-related targets of SMT. Black nodes, myelosuppression-related targets; colored edges, different types of functional interaction between the targets.

Supplementary Tables

Supplementary Table S1. Molecular properties of chemical compounds in SMT.

Herbal medicines	Compounds	MW	OB	Caco-2	DL
AGR	Hemo-sol	136.26	39.84	1.83	0.02
AGR	Scopoletol	192.18	27.77	0.71	0.08
AGR	Decanal	156.30	29.81	1.34	0.02
AGR	beta-Chamigrene	204.39	31.99	1.82	0.08
AGR	Safrol	162.20	45.34	1.44	0.05
AGR	()-alpha-Terpineol	154.28	46.30	1.28	0.03
AGR	o-Thymol	150.24	43.28	1.58	0.03
AGR	D-Galacturonic acid, homopolymer	194.16	29.75	-2.00	0.04
AGR	FERULIC ACID (CIS)	194.20	54.97	0.53	0.06
AGR	nicotinic acid	123.12	47.65	0.34	0.02
AGR	Eucarvone	150.24	53.14	1.35	0.03
AGR	Farnesene	204.39	17.42	1.95	0.05

AGR	(1S,4aR,8aR)-1-isopropyl-7-methyl-4-methylene-2,3,4a,5,6,8a-hexahydro-1H-naphthalene	204.39	19.80	1.86	0.08
AGR	Dodekan	170.38	17.74	1.79	0.02
AGR	bergamotene	204.39	28.51	1.86	0.09
AGR	cuminal	148.22	38.29	1.39	0.03
AGR	Loxanol V	214.44	14.19	1.27	0.05
AGR	Tridecylene	182.39	17.69	1.83	0.03
AGR	80-57-9	150.24	50.63	1.27	0.06
AGR	Usaf hc-1	202.28	16.23	-0.01	0.05
AGR	Azelex	188.25	16.90	-0.04	0.04
AGR	(+)-Ledol	222.41	16.96	1.43	0.12
AGR	Hypnon	120.16	48.19	1.36	0.02
AGR	NON	172.30	26.74	0.96	0.03
AGR	Marmesin	246.28	50.28	0.52	0.18
AGR	()-Cuparene	202.37	38.26	1.88	0.07

AGR	cis-Thujopsene	204.39	56.43	1.84	0.12
AGR	Methylbutenol	86.15	54.58	1.12	0.01
AGR	PCR	108.15	51.99	1.56	0.01
AGR	(4S)-1-methyl-4-(6-methylhepta-1,5-dien-2-yl)cyclohexene	204.39	20.30	1.89	0.06
AGR	Guasol	124.15	51.60	1.28	0.02
AGR	IPH	94.12	36.05	1.50	0.01
AGR	CADINENE	204.39	17.12	1.88	0.08
AGR	Acoradiene	204.39	36.73	1.85	0.07
AGR	Maruzen M	122.18	48.44	1.57	0.02
AGR	o-cresol	108.15	62.45	1.57	0.02
AGR	6,7,3',8'-diligustilide	380.52	9.83	0.79	0.70
AGR	beta-Terpinene	136.26	42.29	1.85	0.02
AGR	Butal	72.12	68.66	1.18	0.00
AGR	Ethol	242.50	13.32	1.31	0.08
AGR	ANN	152.16	29.69	0.69	0.03

AGR	h-Met-h	149.24	70.87	0.06	0.01
AGR	3,4-DIMETHYLBENZALDEHYDE	134.19	39.99	1.40	0.02
AGR	ETHYLBENZALDEHYDE	134.19	40.95	1.40	0.02
AGR	TMHYDROP	152.21	54.42	1.21	0.03
AGR	bicycloelemene	204.39	20.89	1.88	0.08
AGR	WLN: QR CQ DV1	152.16	36.49	0.67	0.03
AGR	WLN: QVR BVQ	166.14	17.74	-0.05	0.04
AGR	(1R,2S,4R)-1-ethyl-1-methyl-2,4-bis(1-methylethyl)cyclohexane	210.45	15.01	1.80	0.06
AGR	DODECENE	168.36	17.74	1.80	0.02
AGR	phosphatdic acid	228.11	19.32	-1.22	0.05
AGR	L-beta,gamma-Dimyristoyl-alpha-cephalin	635.97	20.69	-0.43	0.47
AGR	phosphatidylinositol	390.27	4.63	-2.34	0.29
AGR	phosphatidylinositol_qt	228.11	12.66	-1.15	0.05
AGR	ESEN	148.12	47.31	0.56	0.04
AGR	sedanolide	194.30	62.46	1.24	0.07

AGR	senkyunolide	192.28	68.28	1.28	0.07
AGR	sphingomyelin	493.73	0.31	-0.46	0.51
AGR	Isotetandrine	622.82	10.42	0.95	0.10
AGR	α-acoradiene	204.39	40.98	1.82	0.07
AGR	InChI=1/C15H24/c1-10-7-8-15-9-12(10)14(3,4)13(15)6-5-11(15)2/h7,11-13H,5-6,8-9H2,1-4H	204.39	55.56	1.79	0.10
AGR	α-copaene	204.39	29.33	1.83	0.12
AGR	(1R,4R,5S)-4-isopropenyl-1,8-dimethylspiro[4.5]dec-8-ene	204.39	40.65	1.83	0.07
AGR	2,6-di(phenyl)thiopyran-4-thione	280.43	69.13	1.74	0.15
AGR	o-Xylenol	122.18	53.13	1.62	0.02
AGR	2,4,6-trimethyl-Octane	156.35	29.14	1.81	0.02
AGR	Mesitaldehyde	148.22	37.80	1.54	0.03
AGR	Isoxylaldehyde	134.19	38.85	1.39	0.02
AGR	(E)-octadec-3-ene	252.54	19.50	1.86	0.09
AGR	2-valerylbenzoic acid	206.26	78.26	0.61	0.06

AGR	(Z)-2-Hexenyl hexanoate	198.34	19.39	1.26	0.04
AGR	2-Methylhexadecanoic acid	270.51	20.23	1.07	0.11
AGR	cis-Isoeugenol	164.22	20.73	1.43	0.04
AGR	2-methyl-5-decanone	310.63	20.40	1.42	0.11
AGR	2-methyldodecan-5-one	198.39	13.16	1.43	0.04
AGR	3,7-dimethyl-nonane	156.35	16.97	1.78	0.02
AGR	1,1,5-trimethyl-2-formylcyclohexa-2,5-diene-4-one	164.22	48.94	0.82	0.04
AGR	4-Methyl-6-hepten-3-one	126.22	78.38	1.39	0.01
AGR	6-Ethylresorcinol	138.18	46.45	1.13	0.03
AGR	5-Indolol	133.16	63.14	1.38	0.03
AGR	Undecanol-6	172.35	25.77	1.19	0.02
AGR	7,10-PENTADECADIYNOIC ACID	234.37	41.50	1.32	0.09
AGR	4-chloro-N-[1-methyl-5-[[1-methyl-5-[[1-methyl-5-(2-morpholinoethylcarbamoyl)pyrrol-3-yl]carbamoyl]pyrrol-3-yl]carbamoyl]pyrrol-3-yl]-5-[2-(2-pyridyl)ethylamino]isothiazole-3-carboxamide	762.38	7.18	-0.21	0.31

AGR	Amyl ketone	170.33	18.65	1.31	0.02
AGR	Isoamylbenzene	148.27	35.69	1.84	0.03
AGR	(Z)-2-[[(Z)-2-methylbut-2-enoyl]oxymethyl]but-2-enoic acid	198.24	77.10	0.49	0.04
AGR	Tropone	106.13	47.41	1.25	0.01
AGR	aromadendrene	204.39	18.21	1.83	0.10
AGR	BUA	88.12	21.62	0.69	0.00
AGR	(3S)-3-butyl-3H-isobenzofuran-1-one	190.26	55.05	1.30	0.07
AGR	()-Camphoric acid	200.26	99.13	0.10	0.07
AGR	(3E)-3-butylidene-7-hydroxy-2-benzofuran-1-one	204.24	42.17	1.03	0.08
AGR	Coniferyl ferulate	356.40	4.54	0.71	0.39
AGR	lecithin	678.06	0.31	0.16	0.40
AGR	2,4-Xylylaldehyde	134.19	39.33	1.42	0.03
AGR	m-Ethylphenol	122.18	51.30	1.55	0.02
AGR	1,5,5,6-tetramethyl-1,3-Cyclohexadiene	136.26	39.22	1.82	0.03
AGR	Decursin	328.39	39.27	0.77	0.38

AGR	Lomatin	246.28	23.36	0.48	0.18
AGR/CR	beta-Selinene	204.39	24.39	1.83	0.08
AGR/CR	palmitic acid	256.48	19.30	1.09	0.10
AGR/CR	Nonanal	142.27	40.28	1.31	0.02
AGR/CR	Cymol	134.24	27.20	1.86	0.02
AGR/CR	(-)-alpha-Pinene	136.26	46.25	1.85	0.05
AGR/CR	Myrcene	136.26	24.96	1.84	0.02
AGR/CR	p-Ocimene	136.26	15.06	1.85	0.02
AGR/CR	Moslene	136.26	33.02	1.88	0.02
AGR/CR	CHEBI:7	136.26	45.20	1.84	0.04
AGR/CR	vanillin	152.16	52.00	0.68	0.03
AGR/CR	WLN: VH6	114.21	19.59	1.29	0.01
AGR/CR	o-Acetyl-p-cresol	150.19	24.96	1.02	0.03
AGR/CR	adenine	135.15	62.81	-0.30	0.03
AGR/CR	3-Butylidene-7-hydroxyphthalide	204.24	62.68	1.00	0.08

AGR/CR	Levistolid A	380.52	2.15	0.94	0.82
AGR/CR	Allocymene	136.26	14.89	1.85	0.02
AGR/CR	BdPh	188.24	42.44	1.32	0.07
AGR/CR	senkyunolide-C	204.24	46.80	0.87	0.08
AGR/CR	senkyunolide-D	222.26	79.13	0.12	0.10
AGR/CR	senkyunolide-E	204.24	34.40	0.55	0.08
AGR/CR	(6R)-6-butylcyclohepta-1,4-diene	150.29	31.69	1.85	0.02
AGR/CR	cis-ligustilide	190.26	51.30	1.30	0.07
AGR/PRA	β-Sitosterol	414.79	36.91	1.32	0.75
AGR/RRP	succinic acid	118.10	29.62	-0.44	0.01
AGR/RRP	FER	194.20	39.56	0.47	0.06
AGR/RRP/PRA/CR	Sitogluside	576.95	20.63	-0.14	0.62
RRP	MTL	182.20	17.73	-1.58	0.03
RRP	Arachic acid	312.60	16.66	1.18	0.19
RRP	lauric acid	200.36	23.59	1.02	0.04

RRP	Docosanoate	340.66	15.69	1.21	0.26
RRP	Caffeate	180.17	54.97	0.27	0.05
RRP	Stigmasterol	412.77	43.83	1.44	0.76
RRP	Stachyose	666.66	3.25	-5.54	0.59
RRP	HMF	126.12	45.07	0.05	0.02
RRP	raffinose	504.50	11.79	-3.91	0.66
RRP	leonuride	348.39	2.60	-1.72	0.33
RRP	Daturic acid	270.51	18.51	1.12	0.12
RRP	methyl (2E,4E)-hexadeca-2,4-dienoate	266.47	41.57	1.37	0.12
RRP	zoomaric acid	254.46	35.78	1.18	0.10
RRP	Sumiki's acid	142.12	52.44	-0.21	0.03
RRP	Dihydro-beta-ionone	194.35	26.25	1.35	0.05
RRP	Pca	129.13	96.25	-0.20	0.02
RRP	catalpol	362.37	5.07	-1.72	0.44
RRP	catapol_qt	200.21	44.69	-0.63	0.10

RRP	8-epi-Loganic acid	376.40	4.43	-1.78	0.40
RRP	Forsythiaside	624.65	3.05	-1.92	0.61
RRP	acteoside	624.65	2.94	-1.89	0.62
RRP	2-(4-hydroxyphenyl)ethyl hexacosanoate	516.94	13.12	1.31	0.53
RRP	aeginetic acid	268.39	48.31	0.13	0.13
RRP	Ajugol	348.39	16.87	-1.03	0.32
RRP	Ajugoside	390.43	12.15	-1.03	0.45
RRP	Ajugoside_qt	212.27	81.68	0.24	0.08
RRP	Cerebrosid	266.29	13.81	-2.69	0.11
RRP	Cistanoside A	800.84	3.40	-2.72	0.33
RRP	Cistanoside F	488.49	4.74	-2.11	0.69
RRP	methyl 9,10-methylene-hexadecanoate	282.52	22.94	1.43	0.15
RRP	(2S,3R,4R,5S,6R)-2-[[(1S,4aS,5R,7aR)-4a,5-dihydroxy-7-methylol-5,7a-dihydro-1H-cyclopenta[c]pyran-1-yl]oxy]-6-methylol-tetrahydropyran-3,4,5-triol	362.37	3.10	-2.12	0.37
RRP	melittoside_qt	200.21	40.00	-0.74	0.08

RRP	Dihydrocatalpol	364.39	3.58	-2.24	0.44
RRP	geniposide	388.41	3.78	-2.02	0.44
RRP	geniposide_qt	226.25	39.71	-0.92	0.10
RRP	glutinoside	398.83	21.33	-2.35	0.52
RRP	Jiofuran	184.21	54.91	-0.38	0.06
RRP	jioglutin A	250.70	90.70	0.00	0.13
RRP	jioglutin B	250.70	90.71	-0.01	0.13
RRP	jioglutin C	232.26	2.55	-0.54	0.13
RRP	jioglutin D	246.29	39.02	-0.22	0.14
RRP	jioglutin E	232.31	81.90	-0.04	0.10
RRP	jioglutolide	186.23	86.95	-0.15	0.06
RRP	Jioglutoside A	346.37	3.92	-1.82	0.39
RRP	methyl (1S,4aS,7aS)-7-methylene-1-[(2S,3R,4S,5S,6R)-3,4,5-trihydroxy-6-[[(2R,3R,4R,5S,6R)-3,4,5-trihydroxy-6-methyl-tetrahydropyran-2-yl]oxymethyl]tetrahydropyran-2-yl]oxy-4a,5,6,7a-tetrahydro-1H-cyclopenta[d]pyran-4-carboxylate	518.57	28.27	-1.55	0.84

RRP	Jioglutoside B_qt	210.25	89.22	0.29	0.08
RRP	Jionoside A	800.84	3.62	-2.92	0.36
RRP	Jionoside B	814.87	4.27	-2.53	0.35
RRP	Melittoside	524.53	19.20	-2.94	0.80
RRP	METHYL PALMITOLEATE	268.49	34.61	1.38	0.12
RRP	6-O-p-coumaroylajugol	524.57	26.13	-1.51	0.85
RRP	methyl-2,6,10-trimethyltridecanoate	269.50	24.86	0.85	0.10
RRP	Purpureaside C	786.81	3.14	-2.79	0.38
RRP	Rehmaglutin B	236.67	64.62	-0.55	0.11
RRP	(3aS,4R,6aS)-4-hydroxy-6,6a-dimethylol-3a,4-dihydro-3H-cyclopenta[d]furan-2-one	200.21	29.92	-1.10	0.08
RRP	(2S,3R,4S,5S,6R)-2-[(1R,2R)-2-hydroxy-2-[(E,3S)-3-hydroxybut-1-enyl]-1,3,3-trimethylcyclohexoxy]-6-(hydroxymethyl)tetrahydropyran-3,4,5-triol	390.53	8.43	-1.22	0.33
RRP	(2S,3R,4S,5S,6R)-2-[(1R,2R)-2-hydroxy-2-[(E,3R)-3-hydroxybut-1-enyl]-1,3,3-trimethylcyclohexoxy]-6-(hydroxymethyl)tetrahydropyran-3,4,5-triol	390.53	3.24	-1.21	0.33

RRP	Rehmaionoside C	388.51	12.89	-1.18	0.34
RRP	rehmannioside B	524.53	2.05	-3.18	0.88
RRP	rehmannioside C	510.55	10.23	-2.81	0.86
RRP	6-O-p-hydroxybenzoylajugol	468.50	4.53	-1.66	0.81
RRP	(3R)-2,6,6-trimethyl-3-[(2R,3R,4S,5S,6R)-3,4,5-trihydroxy-6-(hydroxymethyl)oxan-2-yl]oxycyclohexene-1-carboxylic acid	346.42	13.59	-1.28	0.26
RRP	Rehmannioside A	524.53	25.95	-3.00	0.87
RRP	Rehmaglutin A	202.23	29.70	-0.82	0.10
RRP	Rehmaglutin D	220.67	57.03	-0.17	0.10
RRP	6-O-vanilloylajugol	484.50	23.44	-1.26	0.84
RRP	echinacoside	786.81	3.14	-2.91	0.38
RRP	aucubin	346.37	4.17	-1.83	0.33
RRP	Acetylcatalpol	402.44	5.53	-1.57	0.58
RRP/CR	EIC	280.50	41.90	1.16	0.14
RRP/PRA	PENTADECYLIC ACID	242.45	20.18	1.08	0.08

RRP/PRA/CR	Sitosterol	414.79	36.91	1.32	0.75
RRP/PRA/CR	sucrose	342.34	7.17	-2.89	0.23
PRA	PYG	126.12	22.98	0.69	0.02
PRA	Mairin	456.78	55.38	0.73	0.78
PRA	BOX	121.12	31.55	0.54	0.02
PRA	oleanolic acid	456.78	29.02	0.59	0.76
PRA	Kaempferol	286.25	41.88	0.26	0.24
PRA	(+)-Catechin	290.29	54.83	-0.03	0.24
PRA	3,4,5-trihydroxybenzoic acid	170.13	31.69	-0.09	0.04
PRA	Hederagenol	472.78	22.42	0.10	0.74
PRA	Astragalin	448.41	14.03	-1.34	0.74
PRA	TRD	184.41	17.89	1.78	0.03
PRA	(-)-alpha-cedrene	204.39	55.56	1.81	0.10
PRA	DBP	278.38	64.54	0.80	0.13
PRA	EEE	88.12	45.02	1.07	0.00

PRA	Heptadekan	240.53	8.64	1.84	0.07
PRA	LFA	282.62	8.46	1.83	0.13
PRA	Henicosane	296.65	8.41	1.84	0.15
PRA	paeonol	166.19	28.79	0.93	0.04
PRA	Cedrol	222.41	16.23	1.35	0.12
PRA	(6R,10R)-6,10,14-trimethylpentadecan-2-one	268.54	23.30	1.41	0.10
PRA	Pisol	186.38	18.50	1.23	0.03
PRA	Satol	268.54	27.27	1.34	0.11
PRA	(1R)-()-Nopinone	138.23	57.86	1.23	0.05
PRA	myristic acid	228.42	21.18	1.07	0.07
PRA	Octacosane	394.86	8.15	1.91	0.37
PRA	Dodecanal	184.36	21.52	1.40	0.03
PRA	salicylic acid	138.13	32.13	0.63	0.03
PRA	2,2-dimethylcyclohexanol	128.24	82.54	1.22	0.02
PRA	Methyl linolelaidate	294.53	41.93	1.46	0.17

PRA	octadec-9-ene	252.54	19.50	1.87	0.09
PRA	9-methylenefluorene	178.24	26.87	1.95	0.09
PRA	Dipropyl phthalate	250.32	66.30	0.78	0.10
PRA	BU3	90.14	34.87	0.19	0.01
PRA	Bicetyl	450.98	8.03	1.96	0.46
PRA	Dibutylphenol	206.36	38.90	1.73	0.06
PRA	bicyclo[3.1.1]hept-2-ene-2-methanol, 6,6-dimethyl-	152.26	49.79	1.23	0.06
PRA	()-trans-Myrtanol	154.28	49.66	1.17	0.06
PRA	Acetyl oxide	102.10	45.13	0.65	0.01
PRA	ZINC02169908	268.54	23.30	1.42	0.10
PRA	Pulchinenoside A_qt	472.78	16.91	0.12	0.77
PRA	24-Methylenecycloartanol	440.83	10.40	1.42	0.79
PRA	3β,23-dihydroxy-oleana-11,13(18)-dien-28-oic acid	470.76	21.53	0.10	0.75
PRA	3β-hydroxy-11-oxo-olean-12-en-28-oic acid	470.76	13.49	0.21	0.74
PRA	2 - methyl - 3 - (2 - propenyl) - phenol	148.22	52.06	1.64	0.03

PRA	3β-hydroxy-oleana-11,13(18)-dien-28-oic acid?	454.76	17.11	0.55	0.76
PRA	Methylgallate	184.16	30.91	0.26	0.05
PRA	Progallin A	198.19	25.61	0.33	0.06
PRA	(Z)-(1S,5R)-beta-pinen-10-yl-beta-vicianoside	446.55	5.74	-1.56	0.67
PRA	(Z)-(1S,5R)-beta-pinen-10-yl-beta-vicianoside_qt	152.26	50.32	1.52	0.06
PRA	11alpha,12alpha-epoxy-3beta-23-dihydroxy-30-norolean-20-en-28,12beta-olide	470.71	64.77	0.09	0.38
PRA	albiflorin R1	480.51	21.29	-1.53	0.82
PRA	albiflorin R1_qt	318.35	26.18	-0.46	0.34
PRA	propyl (2R)-2-hydroxypropanoate	132.18	25.50	0.44	0.01
PRA	gallotannin	1701.27	7.36	-5.47	0.03
PRA	paeonoside	610.57	3.47	-2.71	0.71
PRA	(3S,3aR,5S,6S,7aR)-5,6-dihydroxy-3,6-dimethyl-3,3a,4,5,7,7a-hexahydrobenzofuran-2-one	200.26	96.64	-0.16	0.07
PRA	Paeoniflorigenone	318.35	87.59	-0.09	0.37
PRA	Palbinone	358.52	43.56	0.00	0.53

PRA	Lactiflorin	462.49	49.12	-1.13	0.80
PRA	2-Hexyl-1-decanol	242.50	17.08	1.29	0.07
PRA	[(3S,3aR,6S,7aR)-6-hydroxy-6-methyl-2,5-dioxo-3a,4,7,7a-tetrahydro-3H-benzofuran-3-yl]methyl benzoate	318.35	17.84	-0.17	0.30
PRA	Paeoniflorin	480.51	53.87	-1.47	0.79
PRA	Paeoniflorin_qt	318.35	68.18	-0.34	0.40
PRA	1,2,3,6-tetra-O-galloylglucose	788.62	3.01	-2.97	0.34
PRA	albiflorin	480.51	12.09	-1.54	0.77
PRA	albiflorin_qt	318.35	66.64	-0.49	0.33
PRA	alexandrin	576.95	20.63	-0.29	0.62
PRA	benzoyl paeoniflorin	584.62	31.27	-0.69	0.75
PRA	4-Chlorobutyric acid	122.56	85.82	0.74	0.01
PRA	galloylpaeoniflorin	632.62	3.03	-1.77	0.42
PRA	oxypaeoniflorin	496.51	21.88	-1.88	0.78
PRA	(3R,3aR,6S,7aR)-6-hydroxy-3,6-dimethyl-3a,4,7,7a-tetrahydro-3H-benzofuran-2,5-dione	198.24	104.94	-0.08	0.08

PRA	(3aR,6S,7aR)-6-hydroxy-6-methyl-3-methylene-3a,4,7,7a-tetrahydrobenzofuran-2,5-dione	196.22	97.79	-0.01	0.08
PRA	cis-5-Octen-1-ol	128.24	31.84	1.16	0.01
PRA	24253-30-3	98.16	74.20	1.33	0.01
PRA	acetic acid	60.06	47.87	0.42	0.00
PRA	Oxypaeoniflorin	496.51	8.38	-1.62	0.78
PRA	10-Methylnonadecane	282.62	10.28	1.84	0.12
PRA	Ethylisobutyrate	116.18	83.67	1.24	0.01
PRA	Pentagalloylglucose	940.72	3.01	-3.08	0.21
PRA/CR	stearic acid	284.54	17.83	1.15	0.14
CR	D-Camphene	136.26	34.98	1.81	0.04
CR	alpha-humulene	204.39	22.98	1.88	0.06
CR	alpha-Curcumene	202.37	4.68	1.93	0.06
CR	2-[(1R,3S,4S)-3-isopropenyl-4-methyl-4-vinylcyclohexyl]propan-2-ol	222.41	19.03	1.37	0.07
CR	PHB	138.13	30.15	0.39	0.03

CR	vanillic acid	168.16	35.47	0.43	0.04
CR	(L)-alpha-Terpineol	154.28	48.80	1.39	0.03
CR	1,8-cineole	154.28	39.73	1.57	0.05
CR	(-)-nopinene	136.26	44.84	1.80	0.05
CR	2-[(2S,5S,6S)-6,10-dimethylspiro[4.5]dec-9-en-2-yl]propan-2-ol	222.41	37.62	1.44	0.09
CR	Furol	96.09	34.35	1.08	0.01
CR	L-Bornyl acetate	196.32	65.52	1.29	0.08
CR	(R)-linalool	154.28	39.80	1.33	0.02
CR	(S)-(+)-alpha-Phellandrene	136.26	27.90	1.87	0.02
CR	-cis-.beta.-Elemene diastereomer	204.39	28.62	1.85	0.06
CR	Methyleugenol	178.25	73.36	1.47	0.04
CR	()-Aromadendrene	204.39	55.74	1.81	0.10
CR	caffeic acid	180.17	25.76	0.21	0.05
CR	L-Limonen	136.26	38.09	1.83	0.02
CR	Tereben	136.26	29.62	1.86	0.02

CR	(1S,5S)-1-isopropyl-4-methylenebicyclo[3.1.0]hexane	136.26	46.21	1.83	0.04
CR	Undekansaeure	186.33	30.14	0.98	0.03
CR	FA	441.45	68.96	-1.50	0.71
CR	()-Terpinen-4-ol	154.28	81.41	1.36	0.03
CR	hexanal	100.18	55.71	1.25	0.01
CR	PENTYLFURAN	138.23	54.59	1.72	0.02
CR	oleic acid	282.52	33.13	1.17	0.14
CR	(R)-(-)-alpha-Phellandrene	136.26	27.51	1.86	0.02
CR	OYA	128.24	19.07	1.30	0.01
CR	o-Cymol	134.24	51.89	1.88	0.02
CR	MYS	212.47	13.98	1.81	0.05
CR	methyl palmitate	270.51	18.09	1.37	0.12
CR	tetradecane	198.44	15.94	1.79	0.04
CR	beta-elemene	204.39	25.63	1.84	0.06
CR	Terpilene	136.26	33.95	1.84	0.02

CR	(R)-p-Menth-1-en-4-ol	154.28	32.16	1.33	0.03
CR	alpha-Farnesene	204.39	21.70	1.97	0.05
CR	.gamma.-Bisabolene	204.39	20.78	1.94	0.06
CR	58870_FLUKA	204.39	49.01	1.82	0.10
CR	Ethylpalmitate	284.54	18.99	1.41	0.14
CR	5-isopropyl-2-methylbicyclo[3.1.0]hex-2-ene	136.26	47.19	1.82	0.04
CR	(1R,5R,7S)-4,7-dimethyl-7-(4-methylpent-3-enyl)bicyclo[3.1.1]hept-3-ene	204.39	16.23	1.86	0.09
CR	(4S)-4-isopropylcyclohexene-1-carbaldehyde	152.26	40.36	1.36	0.03
CR	(S)-2,2,3-Trimethylcyclopent-3-ene-1-acetaldehyde	152.26	45.18	1.32	0.03
CR	WLN: Q1R	108.15	58.68	1.08	0.01
CR	49070_FLUKA	222.41	85.51	1.29	0.12
CR	Mandenol	308.56	42.00	1.46	0.19
CR	germacrene	208.43	15.06	1.82	0.06
CR	METHYL LINOLEATE	294.53	41.93	1.44	0.17

CR	Crysophanol	254.25	18.64	0.62	0.21
CR	uracil	112.10	42.53	0.05	0.02
CR	EUG	150.19	38.39	1.36	0.03
CR	ADO	267.28	15.98	-1.56	0.18
CR	METHYL PENTADECANOATE	256.48	18.82	1.37	0.10
CR	(+)-beta-Phellandrene	136.26	40.30	1.83	0.02
CR	thymol	150.24	41.47	1.60	0.03
CR	alpha-Cubebene	204.39	16.73	1.83	0.11
CR	(+)-ALPHA-FUNEBRENE	204.39	52.87	1.79	0.10
CR	gem-Dimethylcyclopentane	98.21	41.22	1.78	0.01
CR	Senkyunolide-K	208.28	61.75	0.52	0.08
CR	(3Z,6S,7R)-3-butylidene-6-butyryl-7-hydroxy-4,5,6,7-tetrahydroisobenzofuran-1-one	278.38	3.41	0.08	0.16
CR	Senkyunolide-N	226.30	37.27	-0.15	0.10
CR	Senkyunolide-P	382.54	9.38	0.92	0.81

CR	Senkyunolide-Q	278.38	26.84	0.42	0.16
CR	Senkyunolide-R	240.28	13.14	-0.76	0.11
CR	1,1-Diethoxybutane	146.26	29.28	1.30	0.01
CR	Valerophenone	162.25	42.58	1.46	0.03
CR	(1S,5S)-7,7-dimethyl-2-methylenebicyclo[3.1.1]hept-3-ene	134.24	37.71	1.80	0.05
CR	Z-6,8',7,3'-diligustilide	380.52	11.98	0.74	0.70
CR	alpha-Selinene	218.42	31.81	1.82	0.10
CR	1,2,3,4,4a,7-Hexahydro-l,6-dimethyl-4-(1-methylethyl)-naphthalene	162.30	19.03	1.88	0.05
CR	augustic-acid	472.78	21.08	0.18	0.74
CR	m-Ethyltoluene	120.21	50.77	1.87	0.02
CR	(1R,4S,5R)-4-isopropenyl-1,8-dimethylspiro[4.5]dec-8-ene	204.39	40.01	1.85	0.07
CR	β-sesquiphellandrene	204.39	23.68	1.89	0.06
CR	(4aS,7S,8aR)-7-isopropenyl-4a-methyl-1-methylenedecalin	204.39	23.65	1.83	0.08
CR	Artemisia triene	136.26	42.10	1.84	0.02
CR	betea-CUBEBENE	204.39	32.16	1.82	0.11

CR	(1S,4E,8E,10R)-4,8,11,11-tetramethylbicyclo[8.1.0]undeca-4,8-diene	204.39	21.69	1.86	0.08
CR	(Z)-Ligustilide	188.24	53.72	1.30	0.07
CR	Chuanxiongol	218.27	22.19	0.94	0.10
CR	beta-asarone	208.28	35.61	1.45	0.06
CR	cis-Piperitol	154.28	43.26	1.27	0.03
CR	(1S,4R,5R)-1-isopropyl-4-methyl-4-bicyclo[3.1.0]hexanol	154.28	27.22	1.17	0.05
CR	Cnidilide	194.30	77.55	1.21	0.07
CR	1,3,8-p-Menthatriene	134.24	35.86	1.86	0.02
CR	CYCLODODECENE	166.34	47.89	1.84	0.03
CR	cyclohexane,1,1,2,3-tetramethyl-	140.30	48.08	1.77	0.03
CR	2,9-Dimethyldecane	170.38	9.93	1.82	0.02
CR	(2R,4aR)-2-isopropenyl-4a,8-dimethyl-2,3,4,5,6,7-hexahydro-1H-naphthalene	204.39	22.13	1.86	0.08
CR	1,5,5-trimethyl-6-methylenecyclohexene	136.26	46.08	1.83	0.03
CR	Isobutyrophenone	148.22	80.37	1.46	0.03

CR	Myricanone	356.45	40.60	0.67	0.51
CR	neocnidilide	194.30	83.83	1.23	0.07
CR	OCT	114.26	29.72	1.78	0.01
CR	p-Cymen-8-ol	150.24	32.26	1.33	0.03
CR	APH	150.20	24.76	1.17	0.03
CR	Perlolyrine	264.30	65.95	0.88	0.27
CR	PLO	316.53	14.07	0.69	0.43
CR	sedanoic-acid	210.30	44.69	0.37	0.06
CR	senkyunolide-F	206.26	40.35	0.61	0.08
CR	senkyunolide-J	226.30	21.14	0.02	0.10
CR	senkyunolide-L	242.72	29.64	0.63	0.09
CR	senkyunolide-S	240.33	20.61	-0.46	0.11
CR	1-Acetyl-beta-carboline	210.25	67.12	1.18	0.13
CR	Senkyunone	326.52	47.66	1.15	0.24
CR	sinapic acid	224.23	64.15	0.48	0.08

CR	1H-Cycloprop(e)azulen-7-ol, decahydro-1,1,7-trimethyl-4-methylene-, (1aR-(1aalpha,4aalpha,7beta,7abeta,7balpha))-	220.39	82.33	1.37	0.12
CR	trans-2-Nonen-1-ol	142.27	19.96	1.17	0.02
CR	TML	59.13	59.98	1.78	0.00
CR	Wallichilide	412.57	42.31	0.82	0.71
CR	xiongterpene	574.87	23.77	0.44	0.42
CR	1-Octanol,2,7-dimethyl-	158.32	24.43	1.25	0.02
CR	1-terpineol	154.28	49.83	1.24	0.03
CR	1-beta-ethylacrylate-7-aldehyde-beta-carboline	294.33	28.53	0.45	0.31
CR	2-Propionylfuran	124.15	63.12	1.21	0.02
CR	WLN: 2VR	134.19	60.17	1.45	0.02
CR	2,2,3-Trimethylcyclopent-3-ene-1-carboxaldehyde	138.23	42.64	1.31	0.03
CR	methyl 2-pentanoylbenzoate	220.29	69.28	0.91	0.07
CR	ISOHEPTANE	100.23	59.94	1.81	0.01
CR	WLN: T5OJ BVO1	126.12	49.41	1.12	0.02

CR	2-Methyl-1-phenylpropene	132.22	20.17	1.89	0.02
CR	2-methyl-5-(1-methylene)-1,3-cyclohexadiene	106.18	39.91	1.83	0.03
CR	(4S,6S)-cis-Carveol	152.26	32.50	1.22	0.03
CR	2-Methylbenzoxazol	133.16	65.25	1.31	0.03
CR	(5S,6R)-5,6-dimethyltetrahydropyran-2-one	128.19	48.07	1.15	0.02
CR	3(S)-3-Butyl-4,5-dihydrophthalide	194.30	25.76	1.28	0.07
CR	3,4-epoxy-2,2,7,7-tetramethyl-octane	184.36	66.87	1.56	0.05
CR	3-cyclohexen-1-ol	98.16	70.57	1.14	0.01
CR	Methyl 3-furoate	126.12	77.82	1.03	0.02
CR	trans-Piperitol	154.28	47.83	1.19	0.03
CR	4,7-Dihydroxy-3-butylphthalide	222.26	106.09	0.69	0.10
CR	4-iodoindoline	245.07	26.88	1.79	0.03
CR	4-Octanone	128.24	19.37	1.37	0.01
CR	4-hydroxy-3-butylphthalide	206.26	70.31	0.90	0.08
CR	(-)-spathulenol	220.39	25.82	1.45	0.12

CR	5-Propyl-2-thiouracil	170.26	77.60	0.93	0.03
CR	7-oxabicyclo-2.2.1-heptane,1-methyl-4-[1-methylethyl]-	154.28	60.92	1.53	0.04
CR	Aromadendrene oxide 2	220.39	65.10	1.56	0.14
CR	Amylbenzene	148.27	34.34	1.88	0.03
CR	Dimethyl D-malate	162.16	11.47	0.11	0.03
CR	dl-3n-butylphthalide	190.26	47.90	1.30	0.07
CR	Cedrene	204.39	51.14	1.82	0.11
CR	carotol	222.41	149.03	1.46	0.09
CR	Coniferylfcrulate	356.40	4.54	0.67	0.39
CR	Cerulignol	166.24	62.43	1.42	0.04
CR	Decahydro-1,6-bis(methylene)-4-(1-methylethyl)-naphthalene	204.39	28.34	1.84	0.08
CR	Hexaphenone	176.28	19.88	1.49	0.04
CR	(E,E)-1,3,5-Undecatriene	150.29	34.61	1.84	0.02
CR	Heptan	100.23	41.80	1.77	0.00
CR	L-valyl-L-valinc-achydride	214.35	40.18	-0.15	0.05

CR	Levistolide-A	380.52	9.96	0.96	0.82
CR	tetramethylpyrazine	136.22	20.01	1.19	0.03
CR	Exceparl M-OL	296.55	31.90	1.39	0.16
CR	506-43-4	266.52	37.76	1.41	0.12
CR	Octatriacontane	535.16	7.91	1.99	0.37
CR	(2-amylphenyl)methanol	178.30	55.59	1.26	0.04
CR	1(3H)-Isobenzofuranone, 3-butyl-3a,4,5,6-tetrahydro-, cis-(-)-	194.30	65.03	1.25	0.07
CR	Senkyunolide A	192.28	26.56	1.30	0.07
CR	Senkyunolide G	208.28	39.52	0.63	0.08
CR	(3Z,6S,7S)-3-butylidene-6,7-dihydroxy-4,5,6,7-tetrahydroisobenzofuran-1-one	224.28	26.78	0.00	0.10
CR	Germacrene D	204.39	19.22	1.83	0.06

AGR: *Angelicae Gigantis Radix*, RRP: *Rehmanniae Radix Preparata*, PRA: *Paeoniae Radix Alba*, CR: *Cnidii Rhizoma*, MW: molecular weight, OB: oral bioavailability, Caco-2: Caco-2 cell permeability, DL: drug-likeness score.

Supplementary Table S2. Molecular properties of active chemical compounds in SMT.

Herbal medicines	Compound	MW	OB	Caco-2	DL
AGR	Marmesin	246.28	50.28	0.52	0.18
AGR	Decursin	328.39	39.27	0.77	0.38
AGR/PRA	β-Sitosterol	414.79	36.91	1.32	0.75
RRP	Stigmasterol	412.77	43.83	1.44	0.76
RRP/PRA/CR	Sitosterol	414.79	36.91	1.32	0.75
PRA	Mairin	456.78	55.38	0.73	0.78
PRA	Kaempferol	286.25	41.88	0.26	0.24
PRA	(+)-Catechin	290.29	54.83	-0.03	0.24
PRA	11alpha,12alpha-epoxy-3beta-23-dihydroxy-30-norolean-20-en-28,12beta-olide	470.71	64.77	0.09	0.38
PRA	Paeoniflorigenone	318.35	87.59	-0.09	0.37
PRA	Palbinone	358.52	43.56	0.00	0.53
PRA	Paeoniflorin	480.51	53.87	-1.47	0.79

PRA	Paeoniflorin_qt	318.35	68.18	-0.34	0.40
CR	Mandenol	308.56	42.00	1.46	0.19
CR	Myricanone	356.45	40.60	0.67	0.51
CR	Perlolyrine	264.30	65.95	0.88	0.27
CR	Senkyunone	326.52	47.66	1.15	0.24
CR	Wallichilide	412.57	42.31	0.82	0.71

AGR: *Angelicae Gigantis Radix*, RRP: *Rehmanniae Radix Preparata*, PRA: *Paeoniae Radix Alba*, CR: *Cnidii Rhizoma*, MW: molecular weight, OB: oral bioavailability, Caco-2: Caco-2 cell permeability, DL: drug-likeness score.

Supplementary Table S3. List of the targets of active chemical compounds in SMT.

Herbal medicines	Compound	Targets
AGR	Marmesin	ADRB2*, AR*, CDK2*, CHEK1*, CHRM1, CHRM2, DPP4*, ESR1*, F2*, HSP90AA1*, LACTB*, LTA4H, PIK3CG*, PIM1*, PRKACA*, PTGS1*, PTGS2*, PTPN1, RXRA*, SLC6A4*
AGR	Decursin	ACHE*, ADRB2*, ANGPT2*, AR*, CA2*, CALM1*, CDK2*, CHRM1, CHRM3*, CTNNB1*, DAPK3, DPP4*, ESR1*, F2*, HSP90AA1*, KLK3*, MMP9*, PARP1*, PDE3A*, PIM1*, PTGS2*, PTPN1, SCN5A*
AGR/PRA	β-Sitosterol	ADRA1A, ADRA1B*, ADRB2*, BAX*, BCL2*, CASP3*, CASP8*, CASP9*, CHRM1, CHRM2, CHRM3*, CHRM4, CHRNA2, CHRNA7, CYP17A1*, DRD1*, GABRA1, GABRA2, GABRA3 GABRA5, GPBAR1, HSP90AA1*, HTR2A*, ICAM1*, JUN*, KCNH2*, MAP2*, NCOA2*, NPC1L1*, OPRM1*, PARP1*, PDE3A*, PGR*, PIK3CG*, PON1*, PRKACA*, PRKCA*, PTGS1*, PTGS2*, SCN5A*, SLC6A4*, TGFB1*
RRP	Stigmasterol	ABCA1*, ABCG5*, ABCG8*, ADH1C*, ADRA1A, ADRA1B*, ADRA2A, ADRB1, ADRB2*, AKR1B1, CDCA8, CHRM1, CHRM2, CHRM3*, CHRNA7, CTRB1, CXCL8*, CYP17A1*, GABRA1, GABRA3, HTR2A*, IGHG1*, IL10*, LTA4H, LTF*, MAOA*, MAOB*, NCOA1, MDM2*, MEX3D, MTOR*, NCOA2*, NPC1L1*, NR1D2, NR3C2*, PGR*, PLAU*, PRKACA*, PTGS1*, PTGS2*, RAC1*, RUVBL1*, RXRA*, SCN5A*, SLC6A2*, SLC6A3*, SLCO1B1*, TNF*, TNNC1, USF1*
RRP/PRA/CR	Sitosterol	ABCB11*, ABCG5*, ABCG8*, APOE*, CASP3*, CYP17A1*, CYP7A1*, DHCR24, DRAP1, GPBAR1, ICAM1*, NCOA2*, NPC1L1*, NR3C2*, PGR*, RANBP2, SREBF1, SREBF2
PRA	Mairin	AKR1B10*, FOLH1*, GPBAR1, MDM2*, MEX3D, MMAB*, MTOR*, PGR*, POLB*, RAC1*, RNF31*, RPS3*, SAE1, UBA2

PRA	Kaempferol	ABCB1*, ABCC1*, ABCG2*, ACHE*, ADRA1B*, AHR, AHSA1, AKR1B1, AKR1C3*, AKT1*, ALOX5*, AR*, BAX*, BCHE*, BCL2*, CA12, CA2*, CA7, CALM1*, CASP3*, CDK1*, CHRM1, CHRM2, CISD1*, CTDSP1, CYP1A1*, CYP1A2*, CYP1B1*, CYP2D6*, CYP3A4*, DAPK1*, DIO1, DPP4*, EGFR*, ESR1*, ESRRA, F2*, F7*, FLT3*, GABRA1, GABRA2, GSTM1*, GSTM2*, GSTP1*, HAS2, HCK*, HMOX1*, HSD17B1, HSD17B2, HSP90AA1*, ICAM1*, IKBKB*, INSR*, JUN*, MAPK8*, MMP1*, MPO*, NCOA2*, NOS2*, NOS3*, NOX4, NR1I2*, NR1I3*, P4HB*, PGR*, PIK3CG*, PIM1*, PPARG*, PPP3CA, PRKACA*, PRSS1*, PSMD3*, PTGS1*, PTGS2*, PTPRS, RELA*, RPS6KA3, SELE*, SLC2A1*, SLC2A4*, SLC6A2*, SLPI*, STAT1*, TNF*, TOP2A*, TOP2B, TYR*, UGT1A3*, UGT1A7*, UGT1A8*, UGT1A9*, UGT3A1, VCAM1*, XDH*
PRA	(+)-Catechin	ALPL*, APOB*, BACE1*, CA1*, CA12, CA2*, CA3*, CA4*, CA5A, CA5B, CA6, CA7, CA9*, CALM1*, CAT*, CSF2*, DNMT1*, ESR1*, FUT4*, HAS2, HMOX1*, HSP90AA1*, LACTB*, NCOA2*, PGD*, PGF*, PON1*, PRKACA*, PTGS1*, PTGS2*, RXRA*, SLC22A11, SLC47A1
PRA	Paeoniflorigenone	DRAP1, ECH1, FKBP4, GABRA1, HOXB1, NR1D2, RUVBL1*, SULT2A1
PRA	Palbinone	ARHGEF7, MMAB*, NR3C2*, PGR*
PRA	Paeoniflorin	BLVRA, CDY2A, CDY2B, F13A1*, FLI1*, HNRNPR, MAGEA4*, MORF4L18*, SEC23A*
CR	Mandenol	ACBD7, FAAH, GPR174*, GPR34, LPAR1, LPAR2*, LPAR3, LPAR4, LPAR6, NCOA2*, OXER1, P2RY10, PRKCA*, PTGS1*, PTGS2*, TGFA*
CR	Myricanone	ADRB2*, AR*, CCNA2*, CDK2*, CHEK1*, DPP4*, ESR1*, ESR2*, F2*, F7*, GSK3B*, HSP90AA1*, IGHG1*, KCNH2*, KDR*, MAPK14*, NCOA1, NOS2*, PDE3A*, PIM1*, PPARG*, PTGS1*, PTGS2*, RXRA*, SCN5A*
CR	Perlolyrine	AMD1*, F2*, PRKACA*, PTGS2*, PTPRN*, RPS3*, RXRA*, S100A8*
CR	Senkyunone	NR1D2

CR	Wallichilide	BLVRA, HOXB13, NCOA2*, NR3C1*, NR3C2*, PRPS1, PTGS2*, STARD3, VRK2*

AGR: *Angelicae Gigantis Radix*, RRP: *Rehmanniae Radix Preparata*, PRA: *Paeoniae Radix Alba*, CR: *Cnidii Rhizoma*.

*, Targets associated with myelosuppression.
